# The Development of Mismatch Responses to Mandarin Lexical Tone in 12- to 24-Month-Old Infants

**DOI:** 10.3389/fpsyg.2018.00448

**Published:** 2018-04-10

**Authors:** Ying-Ying Cheng, Chia-Ying Lee

**Affiliations:** ^1^Brain and Language Laboratory, Institute of Linguistics, Academia Sinica, Taipei, Taiwan; ^2^Institute of Neuroscience, National Yang-Ming University, Taipei, Taiwan; ^3^Aim for the Top University Project, National Taiwan Normal University, Taipei, Taiwan; ^4^Institute of Cognitive Neuroscience, National Central University, Taoyuan, Taiwan; ^5^Research Center for Mind, Brain and Learning, National Chengchi University, Taipei, Taiwan

**Keywords:** mismatch negativity (MMN), positive mismatch response (P-MMR), infant, lexical tone, Mandarin, event-related potentials (ERPs)

## Abstract

This study explores the development of mismatch responses (MMRs) to Mandarin lexical tone changes in infants at 12, 18, and 24 months of age using the multi-deviant oddball paradigm with the low dipping Tone 3 (T3) as the standard, the high level Tone 1 (T1) as the large, and the high rising Tone 2 (T2) as the small deviant. The results show that the large acoustic change between T1/T3 elicited mismatch negativity (MMN) in all three age groups. The small acoustic change between T2/T3 elicited a positive mismatch response (P-MMR) at 12 and 18 months of age, but no MMR was found to the T2/T3 change at 24 months. The coexistence of MMN and P-MMR in the same age group implies that different mechanisms were used for discriminating large and small deviants. Infants were able to detect the T1/T3 change automatically and showed adult-like MMN as early as 6 months of age. However, the detection of the T2/T3 change remains effortful in infants under 24 months of age. These findings support the notion that MMN and P-MMR may be used to index the maturation of speech perception.

## Introduction

Discriminating ambient phonetic contrasts is an infant’s first step in processing language. Infants’ speech perception has been hypothesized to provide a foundation for future word learning (Werker and Yeung, 2005). For decades, studies on how language experience influences the development of speech perception were mainly focused on consonants and vowels. Both behavioral (Werker and Tees, 1984; Polka and Werker, 1994; Kuhl et al., 2006) and electrophysiological (Cheour et al., 1998b; Rivera-Gaxiola et al., 2005) studies have reported declines in discriminating non-native phonetic contrasts and improvement in discriminating native contrasts between 6 and 12 months of age. Moreover, acoustic characteristics of a contrast also play a role in the developmental timetable (Polka et al., 2001; Sundara et al., 2006; Narayan et al., 2010). The current study aims to explore how acoustic characteristics of a contrast might affect the developmental trajectory of electrophysiological response to Mandarin tonal change in infancy.

As this study involves infants’ electrophysiological responses to Mandarin lexical tones, a description of the Mandarin tones and of Mandarin-learning infants’ and children’s learning of these tones is provided ahead of a review of perception of lexical tones, electrophysiological responses and electrophysiological responses to lexical tones in infancy. Lexical tone is one of the features to determine the meaning of a syllable in Mandarin. There are four lexical tones in Mandarin: the high level tone (T1), the high rising tone (T2), the low dipping tone (T3) and the high falling tone (T4). Studies have shown that children make few errors in producing Mandarin tones at 2 to 3 years of age (Li and Tompson, 1977; Hua and Dodd, 2000; Lin et al., 2008). However, the four tones differ in their acquisition rate: the production of T1 and T4 is mastered earlier than that of T2 and T3. As for perception, pitch height, and contour are crucial for categorizing Mandarin tones (Gandour and Harshman, 1978; Gandour, 1983). Based on the pitch contour, T1, whose F0 remains level over time, is the most distinct from the other three tones, whereas T2 and T3 are acoustically most similar to each other. Tsao (2017) reported that 6- to 8-month-old infants discriminated T1/T3, T2/T3, and T2/T4 contrasts above chance level using the head-turn paradigm. Discrimination performance for the T1/T3 contrast improved in 10- to 12-month-old infants, but discrimination of the other two contrasts did not improve in the same group of infants. Tsao (2008) also showed infants at 12 months of age discriminated the T1/T3 contrast more accurately than the T2/T3 and T2/T4 contrasts. At 3 years of age, children could still easily confuse T3 with T2 in a picture-pointing task (Wong et al., 2005). These findings suggest that the size of acoustic changes could affect the developmental timetable of discriminating lexical tone contrasts from 10 months of age.

Although the perception of vowels and consonants in infancy has been well explored, relatively few studies have investigated the development of lexical tone perception in infancy. Studies across different tonal languages have suggested the phonological representation of lexical tones could attune to ambient language in the first year of life (Harrison, 2000; Mattock and Burnham, 2006; Mattock et al., 2008; Yeung et al., 2013; Cabrera et al., 2015). For example, non-tone language (English and French) infants showed an age-related decline in Thai lexical tone discrimination between 6 and 9 months (Mattock et al., 2008), while tone-language (Mandarin) infants perform equally well at 6 and 9 months for speech (Thai) and non-speech (violin) tone discrimination (Mattock and Burnham, 2006). Yeung et al. (2013) reported that English infants showed declines in Cantonese tone discrimination from 4 to 9 months of age. Moreover, they found that native Mandarin and native Cantonese infants were able to discriminate Cantonese tones at both ages. However, the Mandarin and Cantonese groups showed distinct preferences. Yeung et al. (2013) thus suggested that the perceptual reorganization for lexical tones could begin as early as 4 months of age. Also, the cues used on tone discrimination may change over age. For example, Cabrera et al. (2015) reported that Mandarin and French infants performed equally well in discriminating Mandarin tonal contrasts at 6 months of age, whereas at 10 months of age Mandarin infants relied more on frequency-modulation cues and French infants more relied on amplitude-modulation cues.

In contrast, some studies have shown that the discrimination of lexical tone contrasts in non-tone language infants does not always decline with age (Liu and Kager, 2014; Shi et al., 2017). Dutch infants’ performance in discriminating the Mandarin T1/T4 contrast showed a U-shaped developmental pattern that is infants can discriminate the T1/T4 change at 5–6 and 17–18 months but not at ages in between. Moreover, the rebound of sensitivity is larger when the contrast is acoustically more distinct (Liu and Kager, 2014). In another study, French 4- to 11-month-old infants’ discrimination of the acoustically similar T2/T3 contrast declined with increasing age, but their discrimination of the acoustically less similar T1/T4 contrast remained constant across ages (Shi et al., 2017). Taken together, the extent to which sensitivity to lexical tone contrasts declines in non-tone language infants could depend on the size of the acoustic difference. Tsao (2008, 2017) examined native Mandarin infants’ sensitivity in discriminating Mandarin lexical tones that varied in acoustic similarity. They found that 6- to 8-month-old infants discriminated the acoustically dissimilar T1/T3 contrast and the acoustically similar T2/T3 contrasts equally well. By 10 to 12 months, infants showed improved accuracy in discriminating the T1/T3 contrast but no such improvement for the T2/T3 contrast (Tsao, 2008, 2017). As with the Shi et al. (2017) study, this also suggests that the size of acoustic differences plays a role in the developmental timetable of lexical tone discrimination (Tsao, 2017).

A growing body of studies has used mismatch negativity (MMN), an event-related potential (ERP) component for auditory change detection, to investigate the development of speech perception. Typically, MMN is observed in a passive-oddball paradigm by subtracting ERPs to the standard sounds from that to the deviant sounds. MMN can be elicited without the participant attending to the stimuli. Therefore, it has been widely used in studying auditory perception in infancy. MMN is hypothesized to index automatic change detection when the incoming sound violates the regularity of the previously exposed sequence (Winkler, 2007; Näätänen et al., 2011). MMN amplitude increases and latency decreases as the magnitude of change increases. Furthermore, MMN amplitude can be shaped by the accumulation of language experience in both infancy and adulthood. For example, MMN amplitude to native vowel contrasts increases between 6 and 12 months, while that to non-native contrasts decreases (Cheour et al., 1998b). MMN to Finnish vowel contrasts in fluent Finnish-learning Hungarians is comparable to that of native Finnish speakers, but Hungarians naïve to Finnish did not show an MMN (Winkler et al., 1999). This indicates that MMN could index the development of phonological representation in language acquisition. Therefore, in this study ERP was used as a tool to explore how maturation and the size of acoustic changes might affect the development of neurophysiological responses to Mandarin lexical tone contrasts.

In adults, MMN is typically characterized as a frontally distributed negativity peaking between 100 and 250 ms after the onset of a stimulus. In infants younger than 12 months, studies have reported adult-like MMN to changes in pure tones (Alho et al., 1990; Cheour et al., 2002a,b), durations (Brannon et al., 2004, 2008), vowel contrasts (Cheour-Luhtanen et al., 1995; Cheour et al., 1998a; Kushnerenko et al., 2001; Martynova et al., 2003), and tonal contrasts (Cheng et al., 2013). MMN in infants generally peaks around 300 ms or even later and persists for a longer period compared with MMN in adults. By contrast, other studies have observed a positive mismatch response (P-MMR) between 200 and 450 ms in infants. P-MMRs have also been reported for changes in various features, such as changes in the frequency of pure tones (Leppänen et al., 1997; Morr et al., 2002; Novitski et al., 2007), vowel durations (Friederici et al., 2002), and phonetic contrasts (Dehaene-Lambertz and Dehaene, 1994; Dehaene-Lambertz and Baillet, 1998; Dehaene-Lambertz and Pena, 2001) in newborns and infants younger than 5 months of age. In sum, the polarity and latency of mismatch responses in infancy are highly inconsistent across studies. Therefore, in the following we use MMR as a superordinate term to refer to either MMN or P-MMR found between 100 and 450 ms.

Positive mismatch response has been mainly found in younger infants, especially for smaller deviants. However, the characteristics of P-MMR remain unclear. Studies measuring MMRs across ages have reported P-MMR at 2 to 3 months of age, whereas adult-like MMN is revealed at 4 to 6 months of age and becomes more dominant as the children grow older (Kushnerenko et al., 2002; Trainor et al., 2003; He et al., 2007). Since P-MMRs have mainly been found at younger ages, the presence of P-MMR has been suggested to be related to infants’ maturational status. Other studies have reported that P-MMR tends to be found when it is more difficult to discriminate the change. For example, a smaller pure tone deviant (1000 Hz vs. 1200 Hz) elicits P-MMR in infants younger than 12 months, whereas a larger deviant (1000 Hz vs. 2000 Hz) elicits adult-like MMN from as young as 2 months (Morr et al., 2002). P-MMR has also been found in children as old as 6 to 7 years of age, for smaller deviants (frequency: 1000 Hz vs. 1060 or 1030 Hz; phoneme: “ba” vs. “ta” or “da”) presented with relatively short inter-stimulus intervals (ISI) (Maurer et al., 2003a,b). Similar to MMRs elicited by changes in pure tones, P-MMR to a small deviant and MMN to a large deviant has also been evidenced for phonemic contrasts in 6-month-old infants (Cheng et al., 2013, 2015) and preschoolers (Lee et al., 2012). This suggests that stimuli-related factors, such as short ISI and smaller deviants, also determine the presence of P-MMR.

In addition, studies have shown that P-MMR is more likely to be found in children from disadvantaged language and reading backgrounds. Children with specific language impairment (SLI) required a frequency deviant of more than 10% relative to the 1000 Hz standard to elicit MMN, whereas a deviant of 2–5% is sufficient for the transition from P-MMR to MMN in the age-matched controls (Ahmmed et al., 2008). Also, children with a family history of dyslexia tend to have more positive P-MMRs than their age-matched controls (Maurer et al., 2003a,b). Furthermore, a recent study suggested that the polarity of MMRs could depend on language experience. A relatively difficult English /ta/ vs. /pa/ contrast elicited P-MMR in 11- to 14-month-old infants who had been exposed only to impoverished language input but elicited MMN in an age-matched group who had been exposed to richer language input (Garcia-Sierra et al., 2016). In summary, the presence of P-MMR depends on maturation, the difficulty of the contrasts, the presentation of stimuli, and individual language backgrounds. All these factors should be taken into consideration when investigating the development of MMRs in infancy.

The aim of this study is to systematically explore how maturation and deviant size affect the development of MMRs to lexical tone contrasts in native Mandarin infants. Regarding MMN to lexical tone contrasts, few studies have examined how deviant size affects MMN to Mandarin lexical tones. Cheng et al. (2013) used the acoustically distinct T1/T3 contrast as the large deviant and the acoustically similar T2/T3 contrast as the small deviant and demonstrated that MMN to the T1/T3 contrast has larger amplitude and earlier latency than MMN to the T2/T3 contrast in adults. This finding is congruent with Chandrasekaran et al. (2007), which reported that the deviant size effect on MMN was only found in native Mandarin speakers and not in English speakers without prior experience to a tonal language. Hsu et al. (2014) used the same set of stimuli as Cheng et al. (2013) in a magnetoencephalography (MEG) study to investigate how the deviant size modulates the neural generators underlying the magnetic mismatch response (MMNm) for detecting different magnitudes of lexical tone changes. The more distinct T1/T3 contrast showed larger MMNm in the left hemisphere in comparison with the less distinct T2/T3 contrast. Most critically, the source analysis demonstrated that deviant size affected laterality and the time course of activations in the temporal and frontal cortex. The large deviant showed a greater left-lateralization in superior and middle temporal gyrus. Meanwhile, a set of frontal generators was activated at a later time window to the small deviant, which reflects different top-down mechanisms in responding to large and small deviants (Hsu et al., 2014).

Other studies have also used the same set of stimuli to investigate the developmental trajectories of Mandarin lexical tone perception in preschoolers (Lee et al., 2012) and early infancy (Cheng et al., 2013). The T1/T3 contrast elicited an adult-like MMN in 4-, 5-, and 6-year-olds, but the T2/T3 contrast elicited P-MMR (Lee et al., 2012). The presence of MMN to the T1/T3 contrast suggests that the transition from P-MMR to MMN should occur at a younger age. Cheng et al. (2013) reported that MMRs to the T1/T3 contrast switched from P-MMR in newborns to MMN at 6 months of age. As for the T2/T3 contrast, no significant MMR was found in newborns, and P-MMR was found at 6 months of age. Cheng et al. (2013) suggested that the deviant size effect could be observed in newborns and infants at 6 months of age. However, there is still a gap in empirical evidence between 1 year and 4 years of age.

Meanwhile, other studies with preschoolers and school-age children (Liu et al., 2014; Chen et al., 2016) reported no P-MMR but a late negativity between 385 and 535 ms to the T2/T3 contrast, which the authors likened to an adult-like late discriminative negativity (LDN). Liu et al. (2014) suggested that the single-deviant paradigm used in their study might reduce contextual difficulty in comparison with the multi-deviant oddball paradigm and result in the absence of P-MMR. However, further studies are required to examine this account. LDN is a frontocentrally distributed negativity between 400 and 700 ms after stimulus onset. The LDN has a number of specific qualities as follows. The LDN is predominant in children (Korpilahti et al., 1995; Cheour et al., 2001; Korpilahti et al., 2001; Bishop et al., 2011) and tends to decrease with age (Hommet et al., 2009; Bishop et al., 2011; Liu et al., 2014). In addition, the LDN is more prominent in response to smaller deviants (Bishop et al., 2011) and in children with SLI (Bishop et al., 2010; Kujala and Leminen, 2017), dyslexia (Neuhoff et al., 2012) and attention deficit/hyperactivity disorder (Yang et al., 2015). The LDN is suggested to reflect additional processing for sounds that are difficult to discriminate (Bishop et al., 2011; Liu et al., 2014) and could be associated with higher cognitive functions such as attention-related processing or long-term memory (Neuhoff et al., 2012; Kujala and Leminen, 2017). Chen et al. (2016) reported that a subgroup of 3-year-olds with persistent language delay (PLD) showed a positive MMR to the T2/T3 contrast in 185–335 ms, even though the grand mean of all their 3-year-old participants showed LDN. Taken together, although the ERPs elicited by the T2/T3 contrast are inconsistent across studies, there is a consensus that the T2/T3 contrast does not elicit stable MMN in early childhood. In sum, these studies suggest that the deviant size modulates the polarity of MMR. Given that MMN index a pre-attentive automatic change detection, the transition from P-MMR to MMN for the T1/T3 contrast at 6 months of age suggests that the phonological representation matures for automatically detecting the T1/T3 change by that age. However, the absence of MMN to the T2/T3 contrast suggests that the processing of the less distinct small lexical tone contrasts is still in the process of developing.

The current study aims to further explore the developmental trajectory of MMRs to Mandarin lexical tones from 12 to 24 months by using the same stimuli of Cheng et al. (2013). Given the observation of MMN to the T1/T3 contrast at 6 months in Cheng et al. (2013), an adult-like MMN was expected to be seen from 12 to 24 months of age. As for the T2/T3 contrast, Cheng et al. (2013) reported no MMR in newborns and a P-MMR at 6 months of age. Other studies reported inconsistent findings regarding whether T2/T3 would elicit P-MMR in toddlers (Liu et al., 2014; Chen et al., 2016). Therefore, the developmental trajectory of MMR to T2/T3 contrast and the deviant size effect across ages will be the critical observations in this study.

## Materials and Methods

### Participants

EEG was collected from three groups of infants: 12, 18, and 24 months. For the 12-month-old group, 28 infants attended, but only 14 completed the experiment (3 girls; mean age: 12 months 5 days; range: 11 months 22 days to 12 months 14 days.) For the 18-month-old group, 26 infants attended, of whom 20 completed the experiment (7 girls; mean age: 18 months 5.3 days; range: 17 months 25 days to 18 months 17 days.). As for the 24-month-old group, 29 infants attended, of whom 19 completed the experiment (7 girls; mean age: 24 months 6.4 days; range: 24 months 1 day to 24 months 18 days.). All infants were full-term (gestational age ranged from 37 to 40 weeks) and their parents were native speakers of Mandarin Chinese. All infants passed the otoacoustic emission test for hearing screen at birth. Infants’ cognitive function was assessed using the Bayley Scales of Infant Development-Second Edition: the Mental Developmental Index (BSID-II MDI) before they participated in the EEG recording. Most participants had their BSID-II MDI score within normal range; each group had one infant whose BSID-II MDI score fell in the borderline range (<85 and >70), but none fell below the normal range in their follow-up assessment on the BSID-II MDI 6 months later.

### Design of ERP Experiments

#### Stimuli

The stimuli were the same as those used in Lee et al. (2012) and Cheng et al. (2013). The stimuli consisted of syllables the *yi1* (T1), *yi2* (T2), and *yi3* (T3), which share the same vowel [i] but differ in their pitch contour. Syllable *yi1* (T1) is a high-level tone with the fundamental frequency (F0) around 230 Hz. Syllable *yi2* (T2) is a high rising tone with F0 rising from 180 to 200 Hz. Syllable *yi3* (T3) is a low dipping tone with F0 descending from 100 to 80 Hz and then rising back to 100 Hz. T3 was assigned as the standard; T1 was assigned as the large deviant (level vs. contour); T2 was assigned as the small deviant (contour vs. contour). Stimuli were spoken by a female native speaker of Mandarin and recorded at 16 bits with a sampling rate of 44 kHz. The intensity of the stimuli was normalized to 70 dB, and the duration of stimuli was scaled to 250 ms with Sony Sound Forge 9.0 software.

#### Procedure of Multi-Deviant Oddball Paradigm

During data collection, infants were seated in a high chair or on their caregiver’s lap watching silent cartoons or puppet play to engage them to minimize their movement. The stimuli were presented at a sound pressure level (SPL) of 70 dB through a set of loudspeakers placed approximately 75 cm in front of the infant. The experimental session started with 20 repetitions of the standard (T3) followed by 1000 trials composed of 80% of the standard (T3), 10% of the large deviant (T1), and 10% of the small deviant (T2). The stimuli were presented in a pseudo-randomized sequence, in which at least two standards were presented between any two deviants. In each trial, stimuli lasted for 250 ms with a 500 ms ISI. The whole experiment took about 40 min.

#### EEG Recording and Data Analysis

EEG signals were amplified by NuAmps (Neuroscan Inc.) in direct current (DC) mode, with 100 Hz low-pass and 60 Hz notch filters. Signals were recorded continuously and digitized at a rate of 500 Hz. Signals were recorded from FPz, F3, Fz, F4, C3, C4, O1, O2, and left (M1) and right mastoids (M2) through Ag/AgCl electrodes held with an elastic cap (QuickCap, Neuromedical Supplies, Sterling, VA, United States). Eye movement was monitored with two electrodes attached to the supra-outer canthus of the left eye and infra-outer canthus of the right eye. In the online recording, FPz was considered as ground, and Fz was taken as reference.

For offline processing, the EEG data were re-referenced to the average of M1 and M2. The continuous EEG was segmented into epochs of 800 ms including 100 ms pre-stimulus intervals for baseline correction. A 1 to 20 Hz bandpass filter (zero phase shifting, 12 dB/oct) was applied. Trials with voltage variation exceeding ±100 μV on any electrode were rejected from further analysis. The first 20 standards were excluded from the analysis and only those standards preceded by at least three standards were analyzed to fully control the sequence effect. The grand-averaged ERPs for the standard, the large deviant, and the small deviant were calculated for each participant and electrode. The average number of trials and their standard deviations for each deviant in each age group were: 68.79 (13.55) for T1 and 67.57 (10.75) for T2 in 12-month-old group; 69.9 (17.46) for T1 and 69.5 (17.46) for T2 in 18-month-old group; 72 (12.77) for T1 and 71.89 (12.59) for T2 in 24-month-old group.

Mismatch response is typically distributed at frontal to central sites; therefore, we analyzed electrodes F3, Fz, F4, C3, and C4. To screen the time course of MMRs, we performed a two-tailed paired *t*-test between the standard and each deviant on each sample point in intervals between 100 and 500 ms. Paired *t*-tests were conducted independently at each of the selected sites. MMR was considered meaningful and is reported here when the significant (*p* < 0.05) time points were consecutively longer than 30 ms (Guthrie and Buchwald, 1991). To handle the problems of multiple comparisons, we further examined the identified MMRs by a cluster-based random permutation analysis (Maris and Oostenveld, 2007). First, the consecutive time points with an alpha level less than 0.05 were grouped into clusters. A cluster-level test statistic was calculated by summing all the individual *t*-values within each cluster. Then, computing 1000 randomized cluster-level statistics created a null distribution. Finally, the actual observed cluster-level statistics were compared against the null distribution. If the summed *t*-value of a cluster fell into the highest or lowest 2.5 percentile, the cluster was considered to be significant (alpha < 0.05, two-tailed) in the cluster-based permutation. Clusters with intervals longer than 30 ms and the significance of cluster-based permutations are reported in the following section.

## Results

### MMR at 12 Months

**FIGURE 1:**
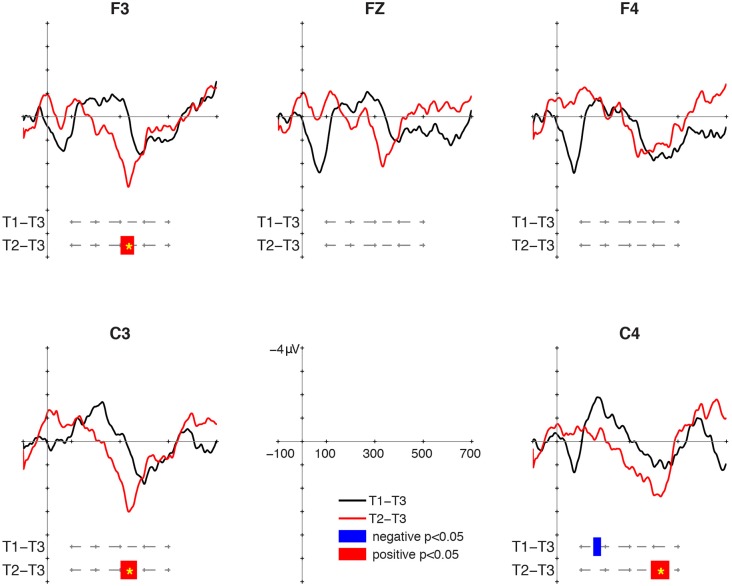
Mismatch responses (MMRs) at 12 months of age. Black lines display the difference wave for the large deviant (LD-S, T1-T3), and red lines display the difference wave for the small deviant (SD-S. T2-T3). Blue bars mark negative clusters, and red bars mark positive clusters in the paired *t*-test. Asterisks in the bars mark those clusters significant in the cluster-based permutation.

Mismatch responses were present but not particularly robust at 12 months of age (**Figure 1**). For the T1/T3 contrast, a negative cluster was found at C4 in the 150–182 ms interval, but it was not significant in the cluster-based permutation (*p* = 0.122). No positive cluster was found for the T1/T3 contrast. As for the T2/T3 contrast, positive clusters were found between 302 and 358 ms at F3, between 302 and 370 ms at C3, and between 388 and 464 ms at C4. The cluster-based permutation showed significant P-MMR to T2/T3 at F3 (*p* = 0.037), C3 (*p* = 0.027), and C4 (*p* = 0.024). The intervals of the clusters and the significance in the cluster-based permutation are summarized in **Table 1**.

**Table 1:** The intervals of positive (P) and negative (N) clusters for each contrast on each site in 12-, 18-, and 24-month-old groups.

		Age		
Contrast	Site	12 months	18 months	24 months
T1/T3	F3		212–278 (N)^∗^	222–274 (N)392–424 (P)
	Fz		214–284 (N)^∗^	218–248 (N)^∗^
	F4		222–288 (N)^∗^	232–278 (N)
	C3		210–250 (N)^∗^	
	C4	150–182 (N)	248–278 (N)	222–296 (N)^∗^
T2/T3	F3	302–358 (P)^∗^	122–170 (P)^∗^ 284–392 (P)^∗^	482–500 (N)^∗^
	Fz		122–162 (P) 310–384 (P)^∗^	
	F4		308–372 (P)^∗^	
	C3	302–370 (P)^∗^	130–162 (P) 286–360 (P)^∗^	
	C4	388–464 (P)^∗^	134–168 (P) 330–388 (P)	

*^∗^p < 0.05 in the cluster-based permutation.*

### MMR at 18 Months

**FIGURE 2:**
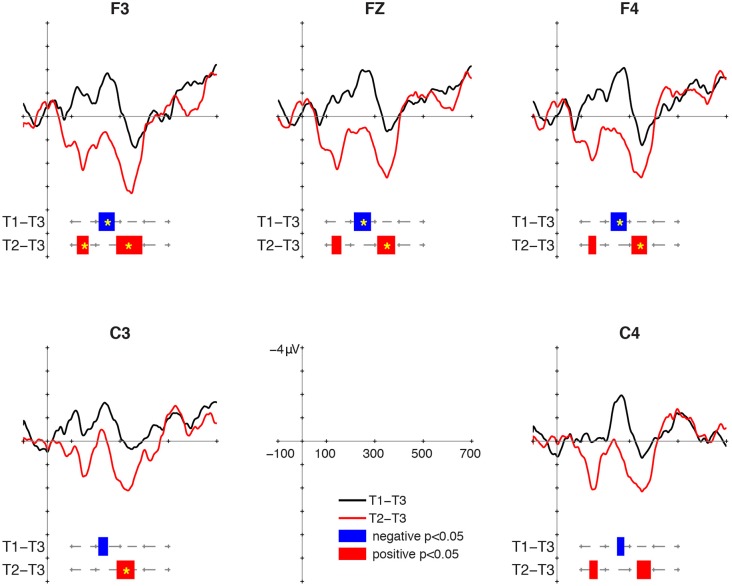
Mismatch responses (MMRs) at 18 months of age.

Mismatch responses to the two contrasts showed distinctly different patterns (**Figure 2**). For the T1/T3 contrast, negative clusters were found at all selected electrodes. Their intervals were 212–278 ms at F3, 214–284 ms at Fz, 222–288 ms at F4, 210–250 ms at C3, and 248–278 ms at C4. The cluster-based permutation showed that the MMN to T1/T3 was significant at F3 (*p* = 0.026), Fz (*p* = 0.039), and F4 (*p* = 0.042). For the T2/T3 contrast, positive clusters were found in two intervals. An early positive cluster was found between 122 and 170 ms at F3, between 122 and 162 ms at Fz, between 130 and 162 ms at C3, and between 134 and 168 ms at C4. The early positive clusters fulfilled the criterion of significance at only F3 (*p* = 0.042) in the cluster-based permutation. In later intervals, positive clusters were found at all selected electrodes. Their intervals were 284–392 ms at F3, 310–384 ms at Fz, 308–372 ms at F4, 286–360 ms at C3, and 330–388 ms at C4. The cluster-based permutation showed significant P-MMR to T2/T3 at F3 (*p* = 0.007), Fz (*p* = 0.025), F4 (*p* = 0.039), and C3 (*p* = 0.033).

### MMR at 24 Months

**FIGURE 3:**
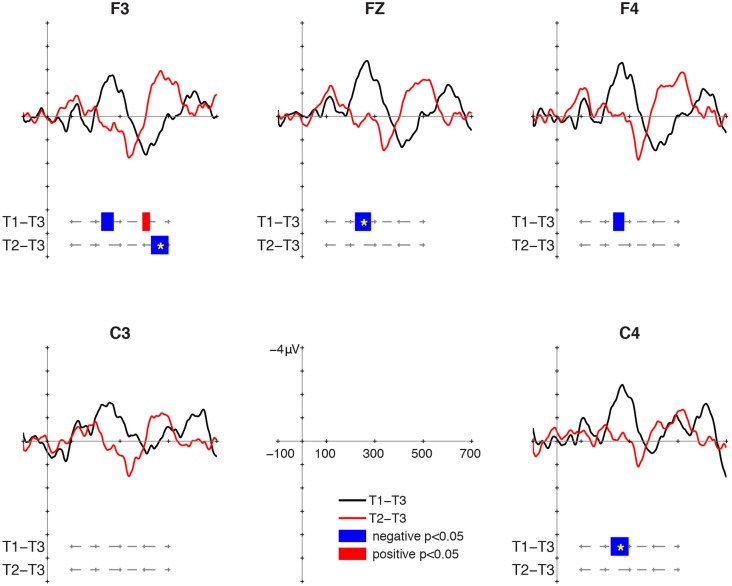
Mismatch responses (MMRs) at 24 months of age.

At 24 months, the T1/T3 contrast elicited MMN. The T2/T3 contrast did not elicit MMR, but a negative cluster was found in intervals later than 450 ms (**Figure 3**). For T1/T3, negative clusters were found in the intervals 222–274 ms at F3, 218–284 ms at Fz, 232–278 ms at F4, and 222–296 ms at C4. The cluster-based permutation showed significant MMN to T1/T3 at Fz (*p* = 0.02) and C4 (*p* = 0.018). Following the MMN, a positive cluster was found for T1/T3 in the interval 392–424 ms at F3, but it was not significant in the cluster-based permutation. The positive cluster was no longer found for T2/T3. Instead, a negative cluster was found for T2/T3 in the 428–500 ms at F3 (*p* = 0.024). This interval was relatively late in comparison with MMR, which suggests that the late negativity might be a component related to change detection but distinct from MMN. The nature of the late negativity for T2/T3 remains unclear, and this will be discussed in the next section.

## Discussion

This study applied the same set of stimuli as Cheng et al. (2013) to explore how deviant size affects the development of MMRs to Mandarin lexical tone discrimination in infants from 12 to 24 months of age. MMN has been well-established in adults to index automatic change detection (Winkler, 2007; Näätänen et al., 2011). However, studies with infants and young children often report a P-MMR, instead of an MMN and demonstrate the developmental change from P-MMR to MMN with age (Kushnerenko et al., 2002; Morr et al., 2002; Lee et al., 2012; Cheng et al., 2013, 2015). Thus, the transition from P-MMR to MMN could serve as a neural marker to index when infants may automatically detect the auditory changes of a set of phonological contrasts. Our findings show that an acoustically large change (T1/T3) elicits MMN in infants at all three ages: 12, 18, and 24 months. In contrast, the acoustically small change (T2/T3) elicits no MMN at any age and a P-MMR in infants at 12 and 18 months of age but not at 24 months. Together the results of this study and that of Cheng et al. (2013) indicate the developmental trajectory of MMR from birth to 24 months of age. The large deviant T1/T3 elicits P-MMR in newborns. This P-MMR transitions into an MMN at 6 months of age and this MMN is sustained at 12, 18, and 24 months of age. As for the small deviant T2/T3, no MMR is found in newborns. The P-MMR appears at 6 months and is sustained at 12 and 18 months but disappears at 24 months. As the T1/T3 and T2/T3 contrasts differ in the pattern of MMRs in all age groups from birth to 24 months of age, it is possible that there are the two types of underlying mechanisms for the discrimination of T1/T3 and T2/T3 contrasts and that these change with development. Although it is unclear whether the absence of MMR at 24 months implies that a transition from P-MMR to MMN would occur at a later age, our current data suggest that infants under 24 months of age are not able to detect the change between T2 and T3 automatically. The potential applications of how the polarity of MMR may be used to index the maturation of lexical tone perception are discussed below.

Following the P-MMR to T2/T3 reported at 6 months of age by Cheng et al. (2013), the current study showed that P-MMR to T2/T3 remained until 18 months and disappeared at 24 months. This is consistent with the idea that P-MMR tends to be found at younger ages (Dehaene-Lambertz and Dehaene, 1994; Friederici et al., 2002; Kushnerenko et al., 2002; Shafer et al., 2011), and to more difficult discriminations, i.e., to smaller deviants (Morr et al., 2002; Lee et al., 2012; Cheng et al., 2013, 2015). Despite the indication from these results that the P-MMR might reflect a less mature speech discrimination process, the functional significance of the P-MMR remains unclear. Nevertheless, given that Tsao (2017) has demonstrated that 6- to 12-month-old infants can discriminate T2/T3 at above chance level, the P-MMR elicited by T2/T3 in the current study might imply a less mature change detection mechanism than for the MMN.

Indeed, the coexistence of MMN to the T1/T3 contrast and P-MMR to the T2/T3 contrast at 12 and 18 months suggests that detecting the two contrasts could depend on different mechanisms. Similar observations for the coexistence of MMNs and P-MMRs have also been reported in other studies with infants (Morr et al., 2002; Friedrich et al., 2009; Cheng et al., 2013, 2015). Friedrich et al. (2004, 2009) suggested that P-MMR could reflect the effort to perceptually categorize the incoming stimuli before the change detection becomes automatic. Our finding of the coexistence of MMN and P-MMR between 6 and 18 months suggests that stimulus-dependent factors might affect whether effortful processes of perceptual categorization or more automatic processes are used for auditory change detection. Besides, the coexistence of MMN and P-MMR is not limited to infancy. It has been found in preschoolers and school-age children (Ahmmed et al., 2008; Lee et al., 2012), especially those who have a history of language and reading disability (Maurer et al., 2003a), and in adults, when a contrast is extremely difficult to discriminate (Kuo et al., 2014). In this latter adult study, it was found that a 1-channel cochlear implant (CI) simulation of the T1/T4 contrast elicited P-MMR in adults with normal hearing, while the natural spoken T1/T4 contrast and the 8- and 32-channel simulations of the T1/T4 contrast elicited MMN. This presence of P-MMR in adults when the spectro-temporal properties of speech sound are drastically degraded supports the idea that P-MMR may index effortful discrimination. Together with Cheng et al. (2013), this series of MMN studies on infant’s lexical tone discrimination show that the T2/T3 contrast elicits no MMR at birth and P-MMR from 6 to 18 months of age. These findings suggest that, for infants under 24 months of age, phonological representations are still developing and are not yet sufficient to automatically discriminate small deviant changes of Mandarin tones.

The current study found two types of deviant size effects. Between 12 and 18 months, the large deviant T1/T3 contrast elicited MMN, but the small deviant T2/T3 contrast elicited P-MMR. Thus, the deviant size effect was reflected in the polarity of MMRs. This pattern is congruent with the deviant size effect on MMRs to lexical tone changes in 6-month-old infants (Cheng et al., 2013), and in 4- to 6-year-old preschoolers (Lee et al., 2012). However, the data show that 24-month-old infants exhibit MMN to the T1/T3 contrast but no MMR to the T2/T3 contrast. So the pattern of deviant size effect turned from the polarity of MMR into the presence or absence of MMN. Other studies have reported the disappearance of MMR in a particular age period. For example, Morr et al. (2002) examined how the deviant size affects the maturation of MMRs to the small (1000/1200 Hz) and large (1000/2000 Hz) frequency changes in infants from 3 to 47 months of age. They found that in infants under 12 months, a small frequency deviant elicited P-MMR, while a large deviant elicited MMN. In other age groups between 13 and 47 months, the large deviant continuously elicited MMN, but no significant MMR was found for the small deviant. The absence of MMR in certain age periods suggests that P-MMR may not transition to MMN immediately, and the time required for the transition from P-MMR to MMN could depend on the discriminability of contrasts. Cheng et al. (2013) reported that MMRs to the more discriminable T1/T3 change switched from P-MMR to MMN between the newborn period and 6 months. The current study showed that the less discriminable T2/T3 contrast elicited P-MMR until 18 months of age, but there was no MMR at 24 months of age, which suggests that brain response to the T2/T3 contrast requires a longer period to transition from P-MMR to MMN than does the brain response to T1/T3. However, the current data are not sufficient to evaluate how long it would take to switch from P-MMR to MMN. Further study is required to determine the age of emergence of MMN to T2/T3.

However, the absence of MMR to T2/T3 in infants at 24 months of age is unexpected, since Lee et al. (2012), using the same experimental design, reported P-MMR to T2/T3 in children between 4 and 6 years of age. Lee et al. (2012) suggested that discrimination of T2/T3 remains effortful in preschoolers. Meanwhile, other studies using the single-deviant paradigm reported no MMR to T2/T3 in children at 3, 5, and 6 years of age (Liu et al., 2014; Chen et al., 2016). One potential account may involve individual differences. Previous studies have suggested that it is more likely to find a P-MMR than an MMN in children with disadvantaged language and reading backgrounds, such as those with SLI (Ahmmed et al., 2008) or a family history of dyslexia (Maurer et al., 2003a,b). Chen et al. (2016) reported no MMR to the T2/T3 contrast in the 185–355 ms interval in 3-year-old children (*n* = 30). However, when they subdivided the children into three groups (PLD, *n* = 10; late bloomer, LB, *n* = 10; and typical language development, TLD, *n* = 10), they found P-MMR for PLD and MMN for TLD children. That is, in grouped results, the MMNs of those who achieve automatic detection (N-responders) may be masked by the P-MMRs of those who still rely on less mature processing (P-responders). Given that Lee et al. (2012) have consistently reported P-MMR in preschoolers from 4 to 6 years, the current absence of P-MMR in the 24-month-old infants may be due to individual variations in their language abilities. Unfortunately, both the current study and Lee et al. (2012) had relatively small sample sizes (*n* = 14∼19 for each age group), too small for subgrouping the participants into different ability groups. Further studies with larger sample sizes and adding behavioral measures of language development are required to explore the proportion of P-responders or N-responders to small deviants in the TLD population. In this way, ERP measures could provide further information about speech perception in early infancy.

In addition, the T2/T3 contrast elicited a late negativity in infants at 24 months of age, with neither P-MMR nor MMN preceding this late negativity. A possible account is that the late negativity may resemble the LDN, which reflects higher cognitive functions for discriminating the T2/T3 contrast. LDN is typically a long-lasting negative deflection from 400 to 700 ms (Korpilahti et al., 1995; Cheour et al., 2001; Korpilahti et al., 2001; Bishop et al., 2011). Yang et al. (2015) used the same set of stimuli as the current study to examine auditory change detection of Mandarin lexical tones in 6- to 12-year-old children with or without ADHD. In response to the T2/T3 contrast, both groups elicited significant LDN from 400 to 700 ms, and neither P-MMR nor MMN preceded this LDN. However, in children between 4 and 6 years of age, the T2/T3 contrast elicited a late negativity in the 385–535 ms interval (Liu et al., 2014; Chen et al., 2016). In the current study, the late negativity for the T2/T3 contrast at 24 months was restricted to between 428 and 500 ms, rather than a long-lasting negative deflection from 450 to 800 ms that is typically shown in the LDN. Taken together, the late negativity elicited by the T2/T3 contrast in children younger than 6 years shows earlier and more restricted latency than the typical LDN does. Whether this late negativity is the typical LDN remains unclear. Another possibility is suggested by a study by Shafer et al. (2011) who found an nMMR, which was a negative component peaking around 340 ms, preceded by a P-MMR in response to English vowel contrast in 3-year-old children. The peak latency of the nMMR shifted approximately 25 ms per year earlier from 3 to 5 years of age (Shafer et al., 2010). Shafer et al. (2010) suggested that the nMMR is an emergence of MMN in the early developmental stage. In the current study, the latency of the late negativity to the T2/T3 contrast found at 24 months resembles that of the nMMR reported in Shafer et al.’s (2010) studies. However, the late negativity in the current study is not preceded by a P-MMR. Taken together, neither LDN nor nMMR can be used to account for the current finding of the late negativity to the T2/T3 contrast at 24 months. Further studies are required to examine whether it resembles LDN or it is an emergence of MMN.

It is worth noting that, MMN in the 12-month-old group was evidenced at only one electrode in a limited interval in contrast to the widely distributed frontal-to-central MMN in the other age groups. When we carefully inspected the status of infants in the EEG collection environment, 12-month-old infants were more likely than those in other age groups to become restless. This 12-month-old group also had a higher rate of attrition (50%) and fewer accepted trials, which could result in less observable MMNs. It is critical to shorten the duration of data collection and improve the quality of data in the future studies of the development of MMN.

## Conclusion

The current study documented MMRs to Mandarin lexical tone discrimination in infants at 12, 18, and 24 months of age. An adult-like MMN to the large deviant T1/T3 contrast was found across all age groups, whereas the small deviant T2/T3 contrast elicited P-MMR at 12 and 18 months of age and no MMR at 24 months of age. These findings suggest that 12- to 24-month-old infants can automatically discriminate T1 and T3, whereas categorization of the acoustically similar tone pair T2 and T3 remains effortful in infants under 24 months of age. In this regard, Kooijman et al. (2013) measured brain responses in a segmentation task in infants at 7 months of age. The majority of 7-month-old infants showed a positive response and a minority showed a left negativity that resembles the responses observed in 10-month-old infants (Kooijman et al., 2005). Critically, these negative responders had higher scores of expressive vocabulary and sentence processing skills at 3 years than did the positive responders, which suggests the polarity of ERP effect may be an important indicator of the maturation of language processing. Taken together, our findings support the notion that the polarity of MMRs may serve as a neural marker to index the maturation of speech perception in infancy.

## Ethics Statement

The study protocols were approved by the Human Subject Research Ethics Committee/Institutional Review Board (IRB) of Academia Sinica, Taiwan. Written consent forms were obtained from parents for their infants’ participation.

## Author Contributions

Y-YC designed and prepared the experiments; acquired, analyzed, and interpreted the data; and drafted and revised this article. C-YL conceptualized this study; supervised and approved the experimental design; interpreted the data; revised the draft; and approved the final version of this article. Y-YC and C-YL agreed to be accountable for all aspects of this study.

## Conflict of Interest Statement

The authors declare that the research was conducted in the absence of any commercial or financial relationships that could be construed as a potential conflict of interest.
